# Comparison of nonhuman primates identified the suitable model for COVID-19

**DOI:** 10.1038/s41392-020-00269-6

**Published:** 2020-10-19

**Authors:** Shuaiyao Lu, Yuan Zhao, Wenhai Yu, Yun Yang, Jiahong Gao, Junbin Wang, Dexuan Kuang, Mengli Yang, Jing Yang, Chunxia Ma, Jingwen Xu, Xingli Qian, Haiyan Li, Siwen Zhao, Jingmei Li, Haixuan Wang, Haiting Long, Jingxian Zhou, Fangyu Luo, Kaiyun Ding, Daoju Wu, Yong Zhang, Yinliang Dong, Yuqin Liu, Yinqiu Zheng, Xiaochen Lin, Li Jiao, Huanying Zheng, Qing Dai, Qiangming Sun, Yunzhang Hu, Changwen Ke, Hongqi Liu, Xiaozhong Peng

**Affiliations:** 1 National Kunming High-level Biosafety Primate Research Center, Institute of Medical Biology, Chinese Academy of Medical Sciences and Peking Union Medical College, Yunnan, China; 2 State Key Laboratory of Medical Molecular Biology, Department of Molecular Biology and Biochemistry, Institute of Basic Medical Sciences, Medical Primate Research Center, Neuroscience Center, Chinese Academy of Medical Sciences, School of Basic Medicine, Peking Union Medical College, Beijing, China; 3 Medical Key Laboratory for Repository and Application of Pathogenic Microbiology, Guangdong Provincial Center for Disease Control and Prevention, Guangzhou, China

**Keywords:** Experimental models of disease, Vaccines

## Abstract

Identification of a suitable nonhuman primate (NHP) model of COVID-19 remains challenging. Here, we characterized severe acute respiratory syndrome coronavirus 2 (SARS-CoV-2) infection in three NHP species: Old World monkeys *Macaca mulatta* (*M. mulatta*) and *Macaca fascicularis* (*M. fascicularis*) and New World monkey *Callithrix jacchus* (*C. jacchus*). Infected *M. mulatta* and *M. fascicularis* showed abnormal chest radiographs, an increased body temperature and a decreased body weight. Viral genomes were detected in swab and blood samples from all animals. Viral load was detected in the pulmonary tissues of *M. mulatta* and *M. fascicularis* but not *C. jacchus*. Furthermore, among the three animal species, *M. mulatta* showed the strongest response to SARS-CoV-2, including increased inflammatory cytokine expression and pathological changes in the pulmonary tissues. Collectively, these data revealed the different susceptibilities of Old World and New World monkeys to SARS-CoV-2 and identified *M. mulatta* as the most suitable for modeling COVID-19.

## Introduction

Coronavirus disease 2019 (COVID-19) caused by severe acute respiratory syndrome coronavirus 2 (SARS-CoV-2) infection was first reported in December 2019. SARS-CoV-2 has become a global pandemic, leading to about 10 million confirmed cases and ~500 thousand deaths. COVID-19 is thought to be the second most widespread infectious disease since the 1918 flu^1^ and has promoted the global collaboration of clinicians and scientists to investigate the diagnosis, prevention and treatment of COVID-19^2^ and to understand genomic and structural information regarding SARS-CoV-2.^3^ However, some challenging and critical questions, including the roles of cytokine storm,^4^ SARS-CoV-2 tropism,^5^ and virus–host interaction,^6^ remain unanswered, and antiviral drugs and vaccines have not been developed,^7^ which requires an animal model that can recapitulate the important features of COVID-19 in patients.^8^ In fact, several animal models of COVID-19 were recently reported; these models include hACE2-transgenic mouse models,^5,9^ a non-transgenic mouse model,^10^ a golden hamster model,^11^ a ferret model^12^ and nonhuman primate (NHP) models.^13–15^ However, in addition to the different genetic backgrounds of the experimental animals, the use of different viral strains, dosages and routes of challenge in these models resulted in different and even controversial conclusions. Therefore, in this study, three species of NHPs were challenged with the same strain of SARS-CoV-2 to identify the suitability of NHP models of COVID-19.

## Results

**Fig. 1:**
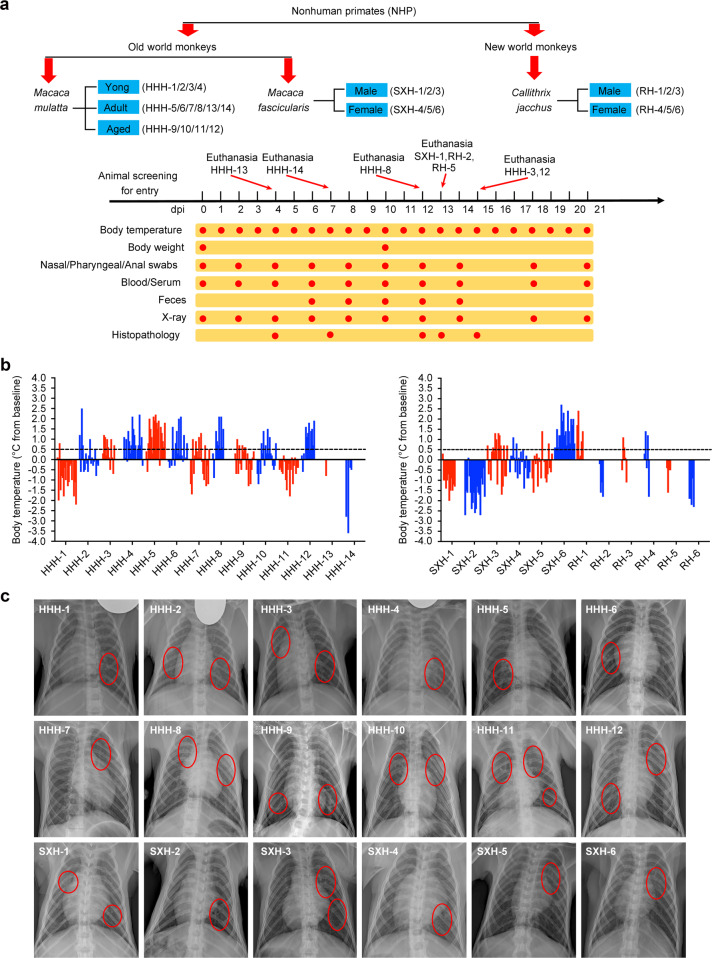
Schematic of the study design and clinical signs of SARS-CoV-2 infection in monkeys. **a** Three species of monkeys from two families of nonhuman primates (26 animals in total) were selected for this comparative study to assess their ability to model COVID-19. Age and sex were considered for grouping of the monkeys. After the collection of baseline samples on day 0, all animals were inoculated with SARS-CoV-2, as stated in the “Materials and methods” section. Clinical signs, viral shedding and replication, and host responses to SARS-CoV-2 were recorded at the indicated time points and evaluated. **b** The body temperature of each monkey was monitored and recorded every two days after SARS-CoV-2 inoculation. The body temperature changes were calculated by subtracting the baseline from each body temperature. The figure was prepared by the Graphpad Prism software. **c** Chest radiographs of SARS-CoV-2-challenged monkeys were taken every two days with a mobile digital medical X-ray photography system. Radiographs were independently graded by two thoracic radiologists in a double-blind manner via a four-pattern approach, as described in the “Materials and methods” section. Abnormal pulmonary sites are marked with red ovals

To determine the suitability of NHP species for modeling COVID-19, we experimentally inoculated three species of monkeys belonging to two families (Old World and New World monkeys) with SARS-CoV-2 (Fig. 1a and Supplementary Table 1) according to the methods described for a reported SARS model,^16^ followed by comprehensive comparisons of the clinical signs of COVID-19, viral shedding and replication, and host responses to viral infection.

### Clinical signs in SARS-CoV-2-infected monkeys

First, a rise in body temperature, one of the clinical signs of SARS-CoV-2 infection, was assessed. Increases in body temperature of more than 0.5 °C were observed in 11 out of 14 *Macaca mulatta* (HHH-1–HHH-14), 4 out of 6 *Macaca fascicularis* (SXH-1–SXH-6) and 3 out of 6 *Callithrix jacchus* (RH-1–RH-6) (Fig. 1b). Notably, *M. fascicularis* SXH-6 showed a continuously high body temperature over the 21-day experimental period. The loss of body weight (BW) was noticed at 10 days post inoculation (dpi), when 11 out of 13 *M. mulatta* showed a decrease in BW by 5.88–28.57%. Five out of six *M. fascicularis* showed a decrease in BW by 2.17–10.51% (Supplementary Table 2), suggesting more severe loss of BW in *M. mulatta* than in *M. fascicularis*.

Next, the effects of SARS-CoV-2 infection on the lung were evaluated on the basis of chest radiographs using a four-pattern approach.^17^ We observed lung abnormalities in the form of nodules, masses, and interstitial patterns in both *M. mulatta* and *M. fascicularis* at 12 dpi. In particular, aged *M. mulatta* showed more interstitial patterning than young or adult *M. mulatta* (Fig. 1c). Importantly, overall comparison of the chest radiographs showed that the pulmonary abnormalities were more severe in *M. mulatta* than in *M. fascicularis*, suggesting that *M. mulatta* are more susceptible to SARS-CoV-2 infection than *M. fascicularis*. However, no other respiratory symptoms were observed in any of the three monkey species during this study.

### Kinetics of SARS-CoV-2 infection in monkeys

**Fig. 2:**
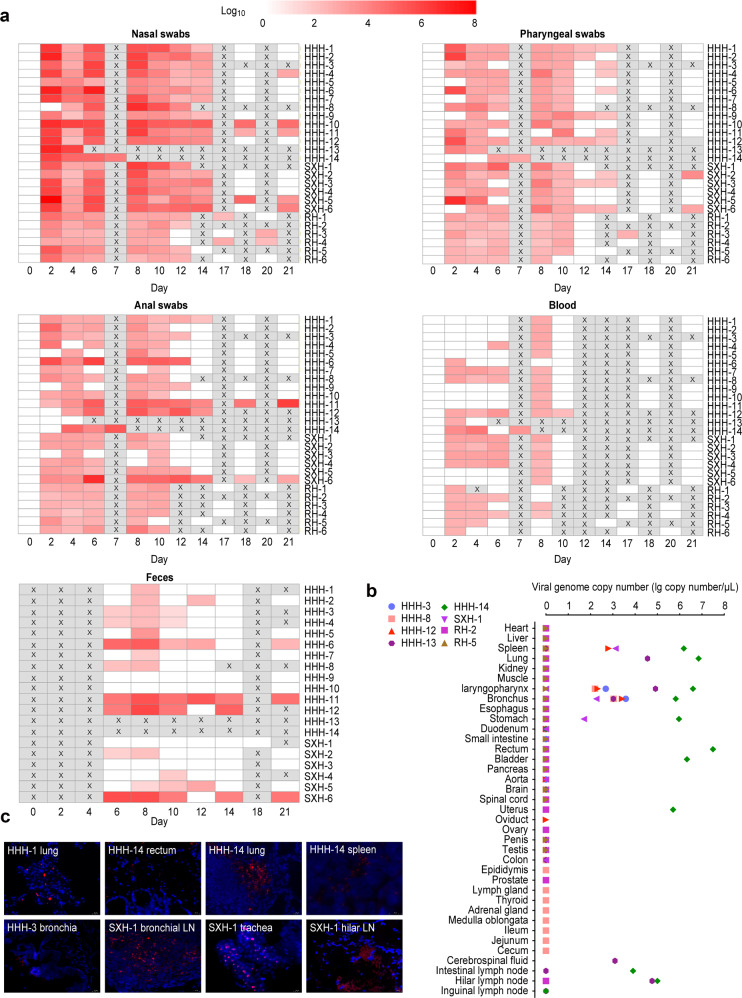
Viral shedding and the replication of SARS-CoV-2 in NHPs. **a** Every other day after virus inoculation, swab (nasal, throat, and anal), feces, and blood samples were collected from the monkeys for the quantification of virus genomic RNA via RT-qPCR. An X in the boxes indicates that the value was not determined. A heatmap was prepared via pheatmap in R package. **b** Tissue samples were harvested from eight necropsied animals at the indicated time points for the evaluation of viral load by RT-qPCR. **c** To determine viral localization in tissues, nucleic acid hybridization (RNAscope technology) was performed to detect viral RNA with a probe specific for SARS-CoV-2

Furthermore, we studied the kinetics of infection by monitoring viral shedding from the respiratory and alimentary tracks and replication in tissues at the indicated time points after infection (Fig. 1a). Nasal swab samples from all three species of monkeys showed peaks in the levels of viral genomic RNA (~ 5.0 × 10^8^ copies/μl) at 2 dpi, followed by two smaller peaks from 6 to 8 dpi. Viral RNA was still detectable in some of the swab samples from Old World monkeys taken at 14 dpi. In contrast, we found lower viral RNA levels in the swab samples from *C. jacchus*. In the peripheral blood, viral RNA levels were very low but detectable in the majority of monkeys at 2 dpi and disappeared from blood samples taken from nearly all monkeys at 10 dpi (Fig. 2a). Moreover, we evaluated the SARS-CoV-2 viral genome in various tissues of the infected monkeys. Viral RNA was detected in the following ten tissues from two *M. mulatta* (HHH-13 and HHH-14) necropsied at the early stage of infection: the lung, trachea, bronchia, spleen, stomach, rectum, bladder, uterus, hilar lymph node, and mesenteric lymph node. However, at the late stage of infection, viral RNA was found in only the bronchus, laryngopharynx, and spleen of *M. mulatta* and *M. fascicularis* (Fig. 2b). Viral RNA and spike protein levels in respiratory tissue samples were confirmed by RNA hybridization with the RNAscope platform (Fig. 2c and Supplementary Table 3) and immunofluorescent assays (Supplementary Fig. 1). We did not find viral RNA in any of the 16–18 tissues collected from the RH2 and RH5 strains of *C. jacchus*. These results suggest that SARS-CoV-2 could replicate in multiple tissues of model *M. mulatta* and *M. fascicularis* and that in addition to the respiratory route, the fecal–oral route may be involved in viral transmission.

### Host responses to SARS-CoV-2 infection

**Fig. 3:**
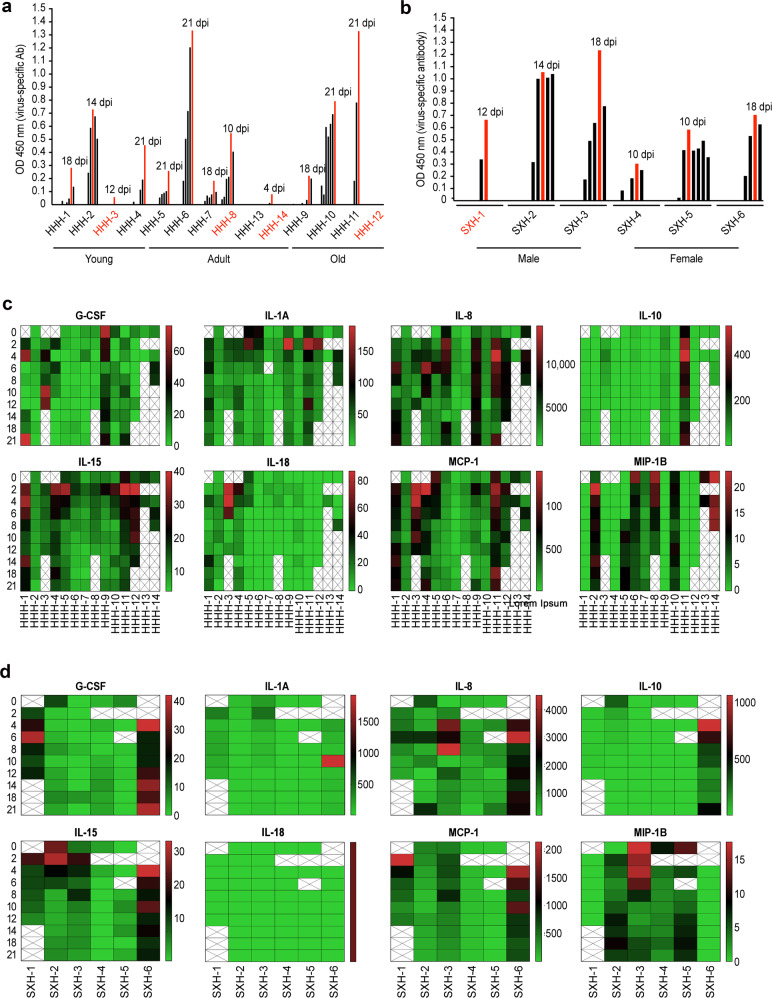
Host responses to SARS-CoV-2 infection. The antibody responses of *M. mulatta* (**a**) and *M. fascicularis* (**b**) to viral infection were evaluated with total virus-specific antibody ELISA kits. Inflammatory cytokines in serum samples from *M. mulatta* (**c**) and *M. fascicularis* (**d**) were measured by Luminex multiplex assays as described in the “Materials and methods” section. All the figures here were prepared via GraphPad software

Finally, host responses to SARS-CoV-2 infection were evaluated by assessments of the production of virus-specific antibodies and secretion of inflammatory cytokines, hematology, and histopathology. We found that SARS-CoV-2 infection induced the production of virus-specific antibodies in the majority of monkeys, with these antibodies becoming detectable at 4 dpi (Fig. 3a, b). Overall, antibody levels in old and adult *M. mulatta* were higher than those in young *M. mulatta*. Consistent with the results of virus monitoring, we did not detect virus-specific antibodies in serum samples from infected *C. jacchus*. Neutralization assays revealed that virus-specific antibodies from most of the animals had neutralizing activity against SARS-CoV-2, with the highest viral titer (1:256) observed in *M. fascicularis* at 21 dpi (Supplementary Table 4).

Inflammatory responses to infection were determined via assays to detect multiple cytokines in serum samples. Eight cytokines (G-CSF, IL-1A, IL-8, IL-15, IL-18, MCP-1, MIP-1B, and sCD40-L) were detected in most of the infected animals. Notably, *M. mulatta* HHH-11 and *M. fascicularis* SXH-6 showed high expression of the inflammatory cytokines IL-10, IL-1A, IL-8, IL-15, and MCP-1 (Fig. 3c, d). Interestingly, the nasal swabs, anal swabs and fecal samples from these two animals exhibited prolonged viral shedding (Fig. 2a), indicating that these inflammatory cytokines were related to SARS-CoV-2 infection. High levels of IL-15, IL-18, and MCP-1 were observed in *M. mulatta* HHH-3, which showed no antibody induction after SARS-CoV-2 infection (Fig. 3c).

Cellular responses to SARS-CoV-2 infection were analyzed by flow cytometry. Three major populations of immune cells (T cells, B cells, and monocytes) in red blood cell (RBC)-depleted peripheral blood were measured. Regardless of the age of *M. mulatta* or sex of *M. fascicularis*, the frequencies of CD4^+^ T cells, CD8^+^ T cells, and monocytes, but not B cells, peaked at 2 or 4 dpi and then gradually declined (Supplementary Fig. 2a, b). The lowest frequencies of these three types of lymphocytes were observed at 10 or 12 dpi, suggesting that SARS-CoV-2 infection caused transient lymphopenia in *M. mulatta* and *M. fascicularis*.

**Fig. 4:**
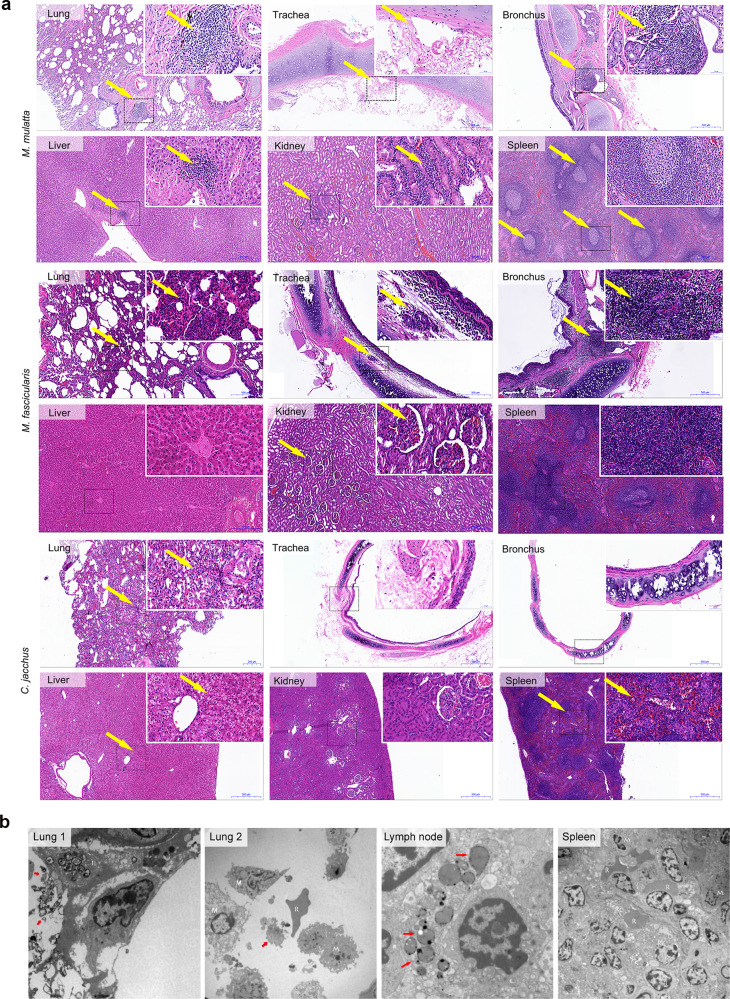
Pathological evaluation of tissues after SARS-CoV-2 infection. **a** After necropsy, tissue samples were cut to the proper size and fixed in 10% neutral buffered formalin for H&E staining, followed by microscopic inspection. Histopathological examination showed thickening of the pulmonary septum and the infiltration of inflammatory cells, diffuse hemorrhage and necrosis in the lungs, and inflammatory infiltration in trachea and bronchus of *M. mulatta*. In the exudate, a small number of lymphocytes and neutrophils were found in some blood vessels of the *M. mulatta* liver, and some hepatocytes were swollen, with multiple vacuole-like cells (mild) and steatosis. The number of germinal centers in the spleen was significantly increased, and capillary hemorrhage occurred in the paracortical area. The renal vesicle space was wider, and some glomeruli had atrophied. In *M. fascicularis*, histopathological changes in the respiratory system were similar to those observed in *M. mulatta*. The kidneys of infected *M. fascicularis* showed the mild infiltration of local inflammatory cells. No histopathological change was observed in liver and spleen. *C. jacchus* showed the slight infiltration of inflammatory cells into the broken pulmonary septum with some necrotic cells. The hepatocytes were swollen. Mild hemorrhage was observed in the spleen, the germinal centers of which were in an actively proliferative condition. No histopathological change was observed in trachea, bronchus and kidney. **b** Tissue samples (from *M. mulatta* HHH-14) fixed in 2.5% glutaraldehyde and a 1% osmium tetroxide solution were subjected to ultrastructural analysis. The red arrow in the Lung 1 section indicates cells in the pulmonary airspace. In the Lung 2 section, M and R indicate macrophages and reticular cells, respectively. In the Lymph node section, the red arrow indicates secretory granules. In the Spleen section, R, Rc and M represent red blood cells, reticular cells, and macrophages, respectively. An increased number of granules was observed in macrophages

More specifically, to determine the pathological responses to SARS-CoV-2 infection, we necropsied eight animals at the early and late stages of infection according to viral load and chest radiograph. Severe gross lesions were observed mainly on the lungs, spleen and lymph nodes dissected from *M. mulatta* and *M. fascicularis* but not *C. jacchus*. The main gross lesions included massive pulmonary punctate hemorrhage, swollen hilar and mediastinal lymph nodes, and swollen mesenteric lymph nodes (Supplementary Fig. 3). Histopathological examination showed thickening of the pulmonary septum and the infiltration of inflammatory cells, diffuse hemorrhage and necrosis in the lung. Mild lesions were also observed in the liver, spleen, and kidney (Fig. 4a). The detailed histopathological changes were described in Fig. 4a. In summary, *M. mulatta* showed pathological changes in all six examined tissues, whereas *M. fascicularis* had four tissues (lung, trachea, bronchus, and kidney) with lesions and *C. jacchus* had pathological changes in three examined tissues (lung, liver, and spleen). Furthermore, typical ultrastructural lesions were observed in the lung (increased numbers of type II pneumocytes, RBCs and macrophages in the alveolar airspace), mesenteric lymph node (intact structure with a large number of lymphocytes, secretory granules in the cytoplasm of cells) and spleen (increased number of granules in the macrophages) (Fig. 4b), which was consistent with the results of microscopic inspection. These results revealed that SARS-CoV-2 infection induced damage to multiple other tissues in addition to the pulmonary organs.

## Discussion

SARS-CoV-2, which remains enigmatic, has rapidly spread worldwide, leading to high economic losses and threats to public health. Within 6 months after SARS-CoV-2 was first reported, five kinds of animals were developed into models in which to study COVID-19: the mouse, ferret, tree shrew, golden hamster, and a NHP species. Murine models of COVID-19 were developed by genetic manipulation of human ACE2 through transgene expression,^9^ CRISPR-cas9 editing,^5^ and the transient overexpression of ACE2,^18^ which makes mice susceptible to SARS-CoV-2 infection. However, due to the many differences in their genetics, anatomy and physiology, these murine models of COVID-19 are not suitable for studies of COVID-19 pathogenesis. The ferret and golden hamster are naturally susceptible to SARS-CoV-2 infection.^11,12^ However, they are also genetically different from humans and are not ideal animal models for studies of vaccines and pathogenesis. The tree shrew is relatively genetically close to primates and can act as an alternative experimental animal to NHPs.^19^ Unfortunately, our recent study showed that the tree shrew is not susceptible to SARS-CoV-2 infection and may instead be an asymptomatic carrier.^20^

NHPs are widely recognized as ideal animal models for emerging and re-emerging diseases, including SARS^16,21,22^ and Middle East respiratory syndrome.^23^ To date, there have been four reports of NHP (Old World monkeys) models of COVID-19^14,15,24,25^ that recapitulate different aspects of COVID-19, but these models have also raised some debate, probably due to differences in animal species (*M. mulatta* and *M. fascicularis*), virus strain, challenge route, and methods of evaluation. Using comparative analysis, our study revealed that *M. mulatta* is a more susceptible species to SARS-CoV-2 infection than *M. fascicularis* and *C. jacchus*.

Host ACE2 is one of the most important receptors for SARS-CoV-2.^26^ At the molecular level, amino acid alignment of the ACE2 fragments that interact with the receptor-binding domain of the SARS-CoV-2 spike glycoprotein revealed the ACE2 fragments from *M. mulatta*, *M. fascicularis*, and humans share the same sequence, while those from *C. jacchus* and humans differ by four amino acids (Supplementary Fig. 4). These four amino acids are critically involved in the binding of ACE2 to SARS-CoV-2.^27^ Y41 and Q42 in hACE2, which bind the S protein via the formation of hydrogen bonds, are substituted by H and E, respectively, in *C. jacchus* ACE2.^28^ These findings may partially explain the lower susceptibility of *C. jacchus* compared to the other species examined to SARS-CoV-2 infection. The sequences of this critical domain in the ACE2 receptors from mouse and ferret differ from that in the hACE2 receptor by eight and seven amino acids, respectively. However, this domain in tree shrew ACE2 differs from that in human ACE2 by ten amino acids. Surprisingly, the golden hamster and human ACE2 receptors differ by only two amino acids. In fact, differences in the number of these amino acids parallel the susceptibility to SARS-CoV-2 infection.

The NHP model in this study recapitulates several clinical features of COVID-19. First, the increased body temperature and abnormal chest radiograph of our model simulate two typical clinical signs in COVID-19 patients, fever and pneumonia, respectively.^29–31^ Importantly, gross lesions of the lungs after necropsy strikingly matched the abnormal spots on chest radiographs taken right before necropsy.

Detection of viral genomic RNA is the most important evidence for the diagnosis of COVID-19 due to SARS-CoV-2 infection. Genomic SARS-CoV-2 RNA is detectable in samples of bronchoalveolar lavage fluid, sputum, nasal swabs, feces, and blood/serum.^32^ Specifically, nasal swabs had higher levels of viral RNA at the early stage of infection for ~2 weeks.^33^ In the present study, the dynamics of viral shedding in the NHP models were similar to those reported in COVID-19 patients. In addition, we noticed a high level of viral shedding via feces that persisted for a long time in four model monkeys (Fig. 2a), suggesting that the fecal–oral route shows great potential risk in the transmission of SARS-CoV-2.^34,35^ The lung is widely considered to be the target organ of SARS-CoV-2 replication since a large viral copy number was detected in bronchoalveolar lavage fluid from COVID-19 patients.^32^ At the early stage of infection, we detected high levels of SARS-CoV-2 viral genomic RNA in the lung and nine other tissues (Fig. 2b, c), including the stomach, rectum, bladder, and uterus, which suggests that SARS-CoV-2 may be transmitted through the fecal–oral, urea, and maternal–fetal routes.^36,37^ Importantly, we also detected viral RNA in the spleen and blood of our model, which is consistent with the clinical findings that SARS-CoV-2 is present in patients’ spleens^38^ and blood and correlated with the severity of COVID-19.^39^

In terms of host responses to SARS-CoV-2 infection, our NHP model partially recapitulates COVID-19 through at least three features. Typical histopathology of pneumonia is observed in COVID-19 patients in additional to mild inflammation in liver.^40^ In addition, secondary lymphoid organs could be directly infected by SARS-CoV-2.^38^ Consistently, our study revealed that after SARS-CoV-2 infection, *M. mulatta* and *M. fascicularis* showed severe histopathological changes in lung, including pneumonia, and inflammation in the spleen and liver (Fig. 4a). The COVID-19-associated cytokine storm is reported to be an important pathogenic factor and a potential promising therapeutic target in COVID-19 patients.^4,41^ Although not consistent with those in COVID-19 patients, the cytokines induced in our model could be used as markers for the evaluation of anti-inflammatory drugs.^42^ Elevated serum IL-6 is clinically correlated with the severity of COVID-19 and regarded as an important biomarker.^43^ In a reported NHP model of COVID-19, the level of IL-6 peaked at 1 dpi and decreased to 0 at 3 dpi.^14^ In this study, we did not detect IL-6, possibly due to our use of a lower challenge dose of virus, which may also have led to the different cytokines profiles and differences in other host responses in the reported NHP models.^14^ By flow cytometric analysis of peripheral blood from *M. mulatta* and *M. fascicularis*, we noticed changes in the abundance of T cells, B cells, and monocytes after viral infection (Supplementary Fig. 2), indicating the induction of host immune responses and transient lymphopenia by SARS-CoV-2 infection.^40^

The NHP models of COVID-19 play critical roles in the research and development of SARS-CoV-2 vaccines. So far, among four NHPs modes of COVID-19 reported, three models are in rhesus monkey (*M. mulatta*) and one in cynomolgus monkey (*M. fascicularis*). Rhesus monkey models are widely used in evaluation of SARS-CoV-2 vaccine. In fact, four SARS-CoV-2 vaccines are all evaluated in rhesus monkeys, including two inactivated vaccines,^44,45^ one adenoviral vectored vaccine^46^ and one DNA vaccine.^47^ Our results in this study further confirmed that rhesus monkey is a good animal model for evaluation of vaccines against SARS-CoV-2.

In conclusion, we have established a NHP model of COVID-19 through comparative analysis of the clinical symptoms, viral replication and tissue tropism, and host responses to SARS-CoV-2 infection among three species from two families of NHPs in one system. Ultimately, this study identified *M. mulatta* as suitable for the study of SARS-CoV-2 infection, as it most closely recapitulated human-like conditions. The use of this model is promising for the development of COVID-19 drugs and vaccines and other basic studies.

## Materials and methods

### Ethics and biosafety statement

All animal procedures were approved by the Institutional Animal Care and Use Committee of the Institute of Medical Biology, Chinese Academy of Medical Science (ethics number: DWSP202002 001), and performed in strict accordance with the guidelines for the National Care and Use of Animals approved by the National Animal Research Authority (PR China) and the Experimental Animal Ethics Committee of the Institute in the ABSL-4 facility of the National Kunming High-level Biosafety Primate Research Center, Yunnan, China.

### Animals and experimental procedures

Three species of monkeys from two primate families (Old World and New World monkeys) used for this study were bred and provided by the Kunming Primate Center of the Chinese Academy of Medical Sciences (Laboratory animal production license# SCXK(Dian)-K2015-0004). The monkeys used in our study were divided into *M. mulatta* group (14 rhesus monkeys, child 4, adults 6, and old 4), *M. fascicularis* group (6 adult monkeys, male 3, and female 3), and *C. jacchus* (6 adult monkeys, male 3, and female 3). All animals were screened for specific pathogens, including SARS-CoV-2, before the experiment (Supplementary Table 1 and Fig. 1a). Throughout this study, animals were fed daily with commercial monkey chow, treat and fruit, and monitored twice a day by the trained personnel. Referencing a NHP model of SARS,^16^ we inoculated adult and aged Old world monkeys with a total of 4.75 ml of 10^6^ pfu/ml SARS-CoV-2 intratracheally (4 ml), intranasally (0.5 ml) and on the conjunctiva (0.25 ml), and a half-dose of the virus was administered to young monkeys. New World monkeys were intranasally challenged with 1.0 ml of the same virus due to their small body size. After viral inoculation, animals were checked daily for clinical signs. Then, we anesthetized the animals with ketamine (6 mg/kg) and performed the following experimental procedures: body temperature and weight monitoring, sample collection, chest radiography, and necropsies at the indicated stages (Fig. 1a).

### Chest radiography

A chest X-ray image of each anesthetized monkey was taken at 55–75 V and 8–12.5 mA every other day using a MobileCooper mobile digital medical X-ray photography system (Browiner, China). Data were independently evaluated in a double-blind manner by two radiologists via a four-pattern approach (analyses of consolidation, interstitial areas, nodules or masses, and atelectasis).

### Hematology

Blood was collected from the inguinal veins of anesthetized animals for flow cytometric analysis conducted on a CytoFLEX instrument (Beckman, USA) using fluorescent-labeled antibodies against the cellular surface markers CD45, CD3, CD4, CD8, CD14, and CD20.

### Quantification of the viral RNA genome

Real-time RT-PCR was used to quantify the viral RNA genome using TaqMan Fast Virus 1-Step Master Mix (Thermo Fisher Scientific, USA) and purified SARS-CoV-2 RNA as a standard curve on a CFX384 Touch Real-Time PCR Detection System (Bio-Rad, USA). Primers and the following probes specific for the *NP* gene were synthesized according to sequences reported by the China CDC: Target-2-F:5′-GGGGAACTTCTCCTGCTAGAAT-3′, Target-2-R:5′-CAGACATTTTGCTCTCAAGCTG-3′, Target-2-P:5′-FAM-TTGCTGCTGCTTGACAGATT-TAMRA-3′. One-step RT-PCR was conducted under the following conditions: 25 °C for 2 min, 50 °C for 15 min, 95 °C for 2 min, and 40 cycles at 95 °C for 5 s and 60 °C for 31 s.

### Histological evaluation

Formalin-fixed paraffin-embedded (FFPE) sections (5 μm) were prepared for hematoxylin and eosin (H&E) staining. The slides were evaluated in a double-blind manner by a pathologist. To prepare ultrastructural sections, tissues were embedded in epoxy resin and cut into 60–70 nm sections. After staining with 2% uranium acetate, ultrathin sections were examined under an H-7650 Hitachi transmission electron microscope (Japan).

### Localization of viral RNA in tissues

A novel nucleic acid hybridization technique, RNAscope, was performed to detect viral RNA in the FFPE sections using a SARS-CoV-2-specific probe (Advanced Cell Diagnostics, USA) designed for NHP tissues. This probe specifically targets the S gene of the SARS-CoV-2 genome. The experiment was conducted according to the procedures provided by the manufacturer.

### Virus-specific antibody

SARS-CoV-2-specific antibodies in serum samples were evaluated via a commercial SARS-CoV-2 Antibody Assay Kit (ELISA) (AnyGo, China) based on the protocol provided by the company.

### Antibody neutralization assay

Serum samples were inactivated at 56 °C for 30 min and diluted 1:8, 1:16, 1:32, 1:64, 1:128, and 1:256 with phosphate-buffered solution. A mixture of SARS-CoV-2 and diluted serum was incubated at 37 °C for 1 h and transferred to Vero E6 cells in a 96-well plate. Cells were continuously incubated at 37 °C for 72 h. Cytopathogenic effects were recorded to evaluate the neutralizing activities of serum samples.

### Multiplex assay of inflammatory cytokines

To assay 23 inflammatory cytokines, the MILLIPLEX MAP NonHuman Primate Cytokine Magnetic Bead Panel—Immunology Multiplex Assay (Millipore, USA) was performed according to the manufacturer’s protocol on a Bio-Plex machine (Bio-Rad, USA). The inflammatory cytokines in this panel are IL-1β, IL-4, IL-5, IL-6, IL-8/CXCL8, G-CSF, GM-CSF, IFN-γ, IL-1ra, IL-2, IL-10, IL-12p40, IL-13, IL-15, IL-17A/CTLA8, MCP-1/CCL2, MIP-1β/CCL4, MIP-1α/CCL3, sCD40-L, TGF-α, TNF-α, VEGF-A, and IL-18.

## Supplementary information

Supplementary Materials for Comparison of nonhuman primates identified the suitable model for COVID-19

## Data Availability

The data sets used for the current study are available from the corresponding author upon reasonable request.
