# Supplementary material for: Comparison of nonhuman primates identified the suitable model for COVID-19
Source: Signal Transduct Target Ther. 2020 Oct 19;5:157. doi: 10.1038/s41392-020-00269-6 (PMC7434851; doi:10.1038/s41392-020-00269-6)
Supplement: Supplementary file 1 — Supplementary Materials for Comparison of nonhuman primates identified the suitable model for COVID-19 [file 41392_2020_269_MOESM1_ESM.docx]

Supplementary Materials for

**Comparison of nonhuman primates identified the suitable model for COVID-19**

Shuaiyao Lu^1,2#^, Yuan Zhao^1#^, Wenhai Yu^1#^, Yun Yang^1#^, Jiahong Gao^1#^, Junbin Wang^1^, Dexuan Kuang^1^, Mengli Yang^1^, Jing Yang^1^, Chunxia Ma^1^, Jingwen Xu^1^, Xingli Qian^1^, Haiyan Li^1^, Siwen Zhao^1^, Jingmei Li^1^, Haixuan Wang^1^, Haiting Long^1^, Jingxian Zhou^1^, Fangyu Luo^1^, Kaiyun Ding^1^, Daoju Wu^1^, Yong Zhang^1^, Yinliang Dong^1^, Yuqin Liu^2^, Yinqiu Zheng^1^, Xiaochen Lin^1^, Li Jiao^1^, Huanying Zheng^3^, Qing Dai^1^, Qiangming Sun^1^, Yunzhang Hu^1^, Changwen Ke*^3^, Hongqi Liu*^1^, Xiaozhong Peng*^1,2^

Correspondence to: pengxiaozhong@pumc.edu.cn; lhq@imbcams.com.cn; kecw1965@aliyun.com

**This PDF file includes:**

Figures. S1 to S4

Tables S1 to S4

**Supplementary Fig. 1**

Immunofluorescent analysis of the SARS-CoV-2 spike protein and ACE2 in the lung, trachea and bronchia from monkeys SXH-1 and HHH-8.

FFPE samples were used for staining. Cellular nuclei were counterstained with DAPI (blue). The viral spike protein was detected with anti-S antibody (red), and the receptor ACE2 was stained with anti-ACE2 antibody (green).

**Supplementary Fig. 2**

Responses of immune cells to SARS-CoV-2 infection.

Percentages of the specific cellular subpopulations in the peripheral blood (PB) of infected *M. mulatta* (a) and *M. fascicularis* (b) were measured by flow cytometric analysis via the cell surface markers CD45, CD3, CD4, CD8, CD20 and CD14. The percentage of positive counts for each immune cell population was defined with the following gating strategies: B cells, CD45^+^CD3-CD20^+^; CD4 T cells, CD45^+^CD3^+^CD4^+^; CD8 T cells, CD45^+^CD3^+^CD8^+^; monocytes: CD45^+^CD14^+^.


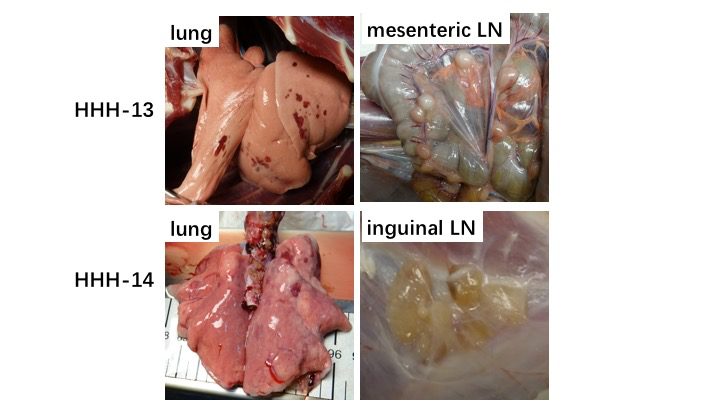


**Supplementary Fig. 3**

Gross lesions were examined and recorded at necropsy.

Representative gross lesions of the lung and lymph nodes from HHH-13 (4 dpi) and HHH-14 (7 dpi) are shown. The main gross lesions included massive pulmonary punctate hemorrhage, swollen hilar and mediastinal lymph nodes, and swollen mesenteric lymph nodes.


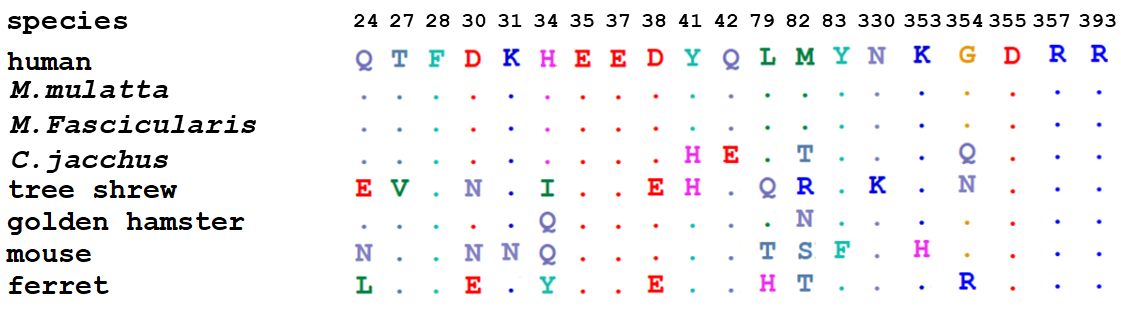


**Supplementary Fig. 4**

Analysis of the binding sites of the ACE2 protein for the RBD of SARS-CoV-2.

Multiple alignment of critical amino acids (AAs) from ACE2 proteins that interact with the RBD of the SARS-CoV-2 spike protein in ACE2 proteins from humans and other mammalian species. The dots indicate amino acids that are identical between human and other mammalian ACE2 proteins. The ACE2 amino acid sequences were obtained from the database Ensembl (*Homo sapiens*: ENSP00000252519; *M. mulatta*: ENSMMUP00000076885; *M. fascicularis*: ENSMFAP00000043589; *C. jacchus*: ENSCJAP00000007838; mouse: ENSMUSP00000073626; ferret: ENSMPUP00000002630; hamster: ENSMAUP00000016335), and the tree shrew ACE2 sequence was obtained from NCBI GenBank (XP_006164754.1).

Table S1.

Animal information

| Species | Gender | Original ID | Label # | Age (yrs) |
| --- | --- | --- | --- | --- |
| *Macaca fascicularis* (6) | Male | 1914053 | SXH-1 | 6 |
|  |  | 1915079 | SXH-2 | 5 |
|  |  | 1915057 | SXH-3 | 5 |
|  | Female | 1814122 | SXH-4 | 6 |
|  |  | 1915002 | SXH-5 | 5 |
|  |  | 1914010 | SXH-6 | 6 |
| *Macaca mulatta* (14) | Young | 19202 | HHH-1 | 1 |
|  |  | 19218 | HHH-2 | 1 |
|  |  | 19185 | HHH-3 | 1 |
|  |  | 19265 | HHH-4 | 1 |
|  | Adult | 15055 | HHH-5 | 5 |
|  |  | 15326 | HHH-6 | 5 |
|  |  | 15046 | HHH-7 | 5 |
|  |  | 15037 | HHH-8 | 5 |
|  |  | 15169 | HHH-13 | 5 |
|  |  | 15170 | HHH-14 | 5 |
|  | Old | 03027 | HHH-9 | 17 |
|  |  | 05114 | HHH-10 | 15 |
|  |  | w1002037 | HHH-11 | 18 |
|  |  | 07160 | HHH-12 | 13 |
| *Callithrix jacchus* (6) | Female | m1 | RH-1 | Adult |
|  |  | m3 | RH-2 | Adult |
|  |  | m5 | RH-3 | Adult |
|  | Male | m2 | RH-4 | Adult |
|  |  | m4 | RH-5 | Adult |
|  |  | m6 | RH-6 | Adult |

Table S2.

Changes of body weight in SARS-CoV-2 infected monkeys

| Species | Groups | Monkey ID | Body weight changes* |
| --- | --- | --- | --- |
| *Macaca fascicularis* | Male | SXH-1 | 2.17% **↓** |
|  |  | SXH-2 | 2.34% **↓** |
|  |  | SXH-3 | 10.51% **↓** |
|  | Female | SXH-4 | 2.31% **↓** |
|  |  | SXH-5 | 0.00% |
|  |  | SXH-6 | 6.67% **↓** |
| *Macaca mulatta* | Young | HHH-1 | 0.00% **↓** |
|  |  | HHH-2 | 5.88% **↑** |
|  |  | HHH-3 | 28.57% **↓** |
|  |  | HHH-4 | 0.00% |
|  | Adult | HHH-5 | 11.43% **↓** |
|  |  | HHH-6 | 10.77% **↓** |
|  |  | HHH-7 | 10.77% **↓** |
|  |  | HHH-8 | 15.12% **↓** |
|  | Old | HHH-9 | 13.04% **↓** |
|  |  | HHH-10 | 12.22% **↓** |
|  |  | HHH-11 | 7.72% **↓** |
|  |  | HHH-12 | 11.36% **↓** |

Table S3.

Summary of viral RNA hybridization via RNAscope.

|  | Non-infected | HHH-13  (4 dpi) | HHH-14  (7 dpi) | HHH-8  (12 dpi) | HHH-3  (15 dpi) | HHH-12  (15 dpi) | SXH-1  (13 dpi) | RH2  (13 dpi) | RH5  (13 dpi) |
| --- | --- | --- | --- | --- | --- | --- | --- | --- | --- |
| Lung | N* | P* | P | N | N | N | N | N | N |
| Trechea | N | N | N | N | N | N | P |  | N |
| Bronchea | N | N | N | N | P | N | N |  | N |
| Spleen | N | N | P | N | N | N | N | N | N |
| Stomach | N | N |  | N | N | N | N | N | N |
| Heart | N | N |  | N | N | N | N | N | N |
| Hillar LN* | N |  |  |  | N | N | P |  | N |
| Bronchial LN |  |  |  |  |  |  | P |  |  |
| Ovary | N |  |  |  |  | N |  | N |  |
| Rectum |  |  | P |  |  |  |  |  |  |

*N means RNA negative.

*P means RNA positive.

*LN means lymph node.

Boxes filled with gray were not performed.

Table S4.

Neutralizing antibodies induced by SARS-CoV-2 infection.

| Species | Original ID | Label# | Days post inoculation | | | | |
| --- | --- | --- | --- | --- | --- | --- | --- |
|  |  |  | 7 | 14 | 21 | 30 |  |
| *M. mulatta* | 19202 | HHH1 | 16* | 32 |  |  |  |
|  | 19218 | HHH2 | 0 | 0 |  |  |  |
|  | 19185 | HHH3 |  | 32 |  |  |  |
|  | 19265 | HHH4 | 8 | 8 |  |  |  |
|  | 15055 | HHH5 | 0 | 8 | 8 | 8 |  |
|  | 15326 | HHH6 | 8 | 16 | 32 | 32 |  |
|  | 15026 | HHH7 | 8 | 16 | 8 | 8 |  |
|  | 15037 | HHH8 | 0 |  |  |  |  |
|  | 03027 | HHH9 | 0 | 32 | 16 |  |  |
|  | 05114 | HHH10 | 0 | 8 | 8 |  |  |
|  | w1002037 | HHH11 | 0 | 16 | 32 |  |  |
|  | 07160 | HHH12 | 8 | 64 |  |  |  |
| *M. fascicularis* | 1914053 | SXH1 | 0 |  |  |  |  |
|  | 1915057 | SXH2 | 128 | 128 | 256 |  |  |
|  | 1915079 | SXH3 | 32 | 64 | 8 |  |  |
|  | 1814122 | SXH4 | 8 | 16 | 8 |  |  |
|  | 1915002 | SXH5 | 8 | 8 | 8 |  |  |
|  | 1914010 | SXH6 | 8 | 128 | 16 | 32 |  |

*Titers of neutralizing antibodies were defined as the maximal dilution of serum that could protect cells from SARS-CoV-2 infection.

Boxes filled with gray were not performed.
